# 2502: mZAP (Zonas, Accion y Proteccion): Empowering communities with mobile strategies for mosquito-borne disease control in tropical environments

**DOI:** 10.1017/cts.2017.151

**Published:** 2018-05-10

**Authors:** Jose G. Perez-Ramos, Scott McIntosh, Carmen M. Velez Vega, Emily S. Barrett, Timothy De Ver Dye

**Affiliations:** University of Rochester Medical Center, Rochester, NY, USA

## Abstract

OBJECTIVES/SPECIFIC AIMS: Our objectives with this project are to engage communities through technology creating a communication channel with affected communities and stakeholders about mosquito-borne illness, vector control and environmental health risk. Furthermore, engaging communities to electronically map ecological risks that impact mosquito-borne illness with the goal of creating a mobile application that will work as an ecological surveillance against mosquito proliferation and potential mosquito population reduction, and finally pilot test and evaluate potential benefits in communities where the application was used. METHODS/STUDY POPULATION: We propose a methodology to perform formative community work that will underscore a distributed, democratized ecological surveillance through an integration of multidimensional health behavior theories that address the challenges of ZIKV in Culebra, a marginalized island community off the coast of the main island of Puerto Rico. Using participatory design, we will develop, test, and evaluate users’ experiences towards mobile applications using qualitative (interviews) and quantitative (survey) methodologies. A mobile application with the capacity of mapping, use of social-media, crowdsourcing, and photo-voice in a dynamic and simple way will allow community members to alert “hot-zone” locations to the stakeholders interested in creating ecological action in their community. This multidimensional concept integrates explanatory and prospective approaches and will generate systematic short-term solutions for mosquito control and long-term solutions providing the necessary tools for community empowerment. RESULTS/ANTICIPATED RESULTS: Our proposed design will facilitate better understanding of the interactions between community members and socio-environmental determinants of mosquito-borne diseases. Furthermore, our proposed project will not only facilitate communication among members of a community, but also it will provide a platform for engagement and empowerment, establishing a change in the preventive paradigm of how communities face the negative impacts of micro-ecologies that surround them. DISCUSSION/SIGNIFICANCE OF IMPACT: Our proposed community collaboratory mHealth tool mZAP! (Zonas, Accion y Proteccion) will address the lack of community participation efforts against mosquito-borne diseases contributed simultaneously by the disengagement and disempowerment of community members. mZAP! will serve as an innovative tool to engage marginalized and communities made vulnerable in Puerto Rico. This approach should be successful as Puerto Rico is one of the most digitally connected countries in Latin America, with high mobile phone usage rates and social media use. Using mZAP!, communities will report and map breeding sites, use social media and crowd sensing, targeting against powerful tools against mosquito ecologies in their own environments. This application could result in an effective way to change the paradigms for public health approaches to use Information Communications Technologies (ICTs) to empower communities.

